# Dealing with Ethical Concerns in Suicide Research: A Survey of Australian Researchers

**DOI:** 10.3390/ijerph16071094

**Published:** 2019-03-27

**Authors:** Karl Andriessen, Lennart Reifels, Karolina Krysinska, Jo Robinson, Georgia Dempster, Jane Pirkis

**Affiliations:** 1 Centre for Mental Health, Melbourne School of Population and Global Health, The University of Melbourne, Parkville, VIC 3010, Australia; karl.andriessen@unimelb.edu.au (K.A.); l.reifels@unimelb.edu.au (L.R.); karolina.krysinska@unimelb.edu.au (K.K.); georgia.dempster@unimelb.edu.au (G.D.); 2 Orygen, the National Centre of Excellence in Youth Mental Health, Parkville, VIC 3052, Australia; jo.robinson@orygen.org.au

**Keywords:** ethical review, ethics, research, research ethics, suicide, prevention

## Abstract

Given the increasing trend in suicide mortality and its burden on individuals, families and communities, ethically sound research is crucial to improve the prevention of suicidal behaviour. However, few studies have looked at the experiences of researchers in obtaining ethics approval for their studies. This study addressed this gap by investigating researchers’ experiences in obtaining ethics approval and how they dealt with the concerns raised by ethics committees. Respondents were recruited from September to November 2018 through the Australian Suicide Prevention Research Leaders Network, and 33 respondents (35%) completed the study survey, comprising forced-choice and open-ended questions. Respondents most commonly reported concerns from ethics committees regarding potential harm to participants and researchers’ responsibilities to participants within the context of intervention and evaluation studies. Most researchers modified their ethics application and/or consulted with their ethics committee to reply to the concerns raised. Most respondents perceived the impact of the modification as positive or neutral. The study concludes that researchers may anticipate potential concerns of ethics committees. Improved understanding of how ethics committees work and dialogue between researchers and ethics committees should sustain the quality in suicide-related research.

## 1. Introduction

Suicide constitutes a major public and mental health problem in Australia. Over the last ten years the annual number of suicides has increased from 2341 in 2008 to 3128 in 2017 [1]. This represents an increase of the age-standardized suicide rate (per 100,000 persons) of 10.9 in 2008, to 12.6 in 2017 [1], exceeding the global age-standardized suicide rate of 10.5/100,000 persons [2]. In addition to the lives lost to suicide, annually, an estimated 0.4% of the Australian population (males: 0.3%, females: 0.5%) attempt suicide, and about 4.3% of the population are exposed to a suicide death in a given year [3,4]. Both those who have attempted suicide, or have been bereaved by suicide, have increased risks of social and mental health problems and suicidal behaviour [5].

Given the increasing trend in suicide mortality and the burden on individuals, families and communities, high quality research is crucial to improve the prevention of suicidal behaviour, and to better help suicidal individuals and those affected by suicide. However, the low base rate of suicide and the impossibility of predicting the occurrence of suicide in individuals are major challenges in conducting suicide prevention research [6]. Also, the thresholds used to balance ethical issues in suicide-related research may differ between researchers and ethics committees or institutional review boards. For example, researchers may wish to include individuals with considerable levels of suicide risk in intervention studies, as excluding these participants will preclude the assessment of the effectiveness of intervention for those most in need [7,8]. Conversely, ethics committees may require that these participants are excluded from such studies based on concerns for their safety. Similarly, ethics committees may hold the view that asking participants questions about suicidal behaviour or suicide bereavement may increase their risk of non-fatal or fatal suicidal behaviour, despite evidence to the contrary [9,10]. Furthermore, an exercise in balancing safety issues may be compounded by (underlying) issues stemming from moral views on suicide, related to the (un-) acceptability of suicide and the moral obligations of an individual to save a life [11].

The National Health and Medical Research Council (NHMRC) National Statement on Ethical Conduct in Human Research 2007 (Updated 2018) is a document used by researchers, ethics committees, governance committees and research participants as a guiding ethos for human research [12]. The National Statement has identified four core values: respect for human beings (including guarding autonomy), research merit and integrity, justice (benefits and burdens of research, and procedural justice in ‘fair treatment’), and beneficence (assessing and taking account of the potential harm and benefits of research to participants and to the wider community). The National Statement further stipulates that the responsibility for the ethical design and conduct of human research is exercised at various levels by researchers, ethics committees, funding organizations and governments [12].

Given the high need for and the sensitive nature of suicide-related research, surprisingly few studies have looked at the experiences of researchers in obtaining ethics approval for their studies. Our searches of the literature identified only one study conducted ten years ago with a sample of 28 researchers who were predominantly from the United Kingdom and the United States [13], and 125 members of international ethics committees [14]. Most researchers in this study stated that they had experienced few problems in obtaining ethics approval, possibly due to their careful anticipation of potential concerns related to accessing the study population, their maintenance of confidentiality and responsibility of care for participants [13]. However, some researchers reported that they had encountered resistance from their ethics committee to conducting suicide-related research. Conversely, potential harm to participants, responsibility of the researcher to participants, and participant competency and consent were the major concerns raised by the ethics committees, and some committee members stated that, in their view, ethics committees tended to be paternalistic [14].

Responding to the increasing trend in suicide mortality, the Australian Government [15] has invested in concerted suicide prevention and research. Our team has received funding specifically to conduct the National Leadership in Suicide Prevention Research (NLSPR) project. Started in 2018, the project currently comprises four interrelated work streams focused on understanding needs, identifying effective interventions, exchanging knowledge and building capacity [16]. The project aims to inform a future suicide prevention research agenda, and strengthen the suicide prevention research and evaluation capacity of key stakeholders [16].

An important priority of the NLSPR project was to investigate the experiences of Australian researchers in obtaining ethics approval for suicide-related research, and how they dealt with the concerns raised by ethics committees. This study specifically investigated the following questions: Which institutions’ ethics committees have researchers submitted ethics applications to? What kind of concerns have been raised by those ethics committees in response to the applications? How have researchers dealt with these concerns? How has this impacted, if at all, on the research? Based on the findings of this study, our team will further investigate the experiences of Australian ethics committees in dealing with suicide-related ethics applications. The overarching aim of our study was to improve understanding in this area, with the objective of supporting researchers and ethics committees to make decisions that minimize risks and maximize benefits, and to better facilitate safe and ethical research into suicide prevention. The study received clearance from the University of Melbourne’s Human Research Ethics Committee on 23 August 2018 (Ethics ID 1852648.1). In this paper, ‘respondents’ are the researchers who participated in our survey, and ‘participants’ are those who participated in the respondents’ studies or in research in general.

## 2. Materials and Methods

### 2.1. Survey

We created an online survey for this study which included forced-choice and open-ended questions. Questions regarding type, focus and setting of the study in the ethics application were based on previous research of our group [16]. Questions regarding respondents’ experiences with ethics applications were based on the literature (e.g., [13]) and topics addressed in the NHMRC National Statement on Ethical Conduct in Human Research 2007 (Updated 2018) [12].

The survey inquired about how long respondents had worked in suicide-related research, how many ethics applications they had submitted for suicide-related studies over the last five years, and what the outcomes of these applications were (e.g., minor/major revision). It also asked respondents to identify the ethics committee which had considered most of their applications. Next, the survey asked respondents to focus on one ethics application where concerns had been raised, and to answer questions regarding the type of study, the study population, the setting, any concerns raised, how they dealt with them and the impact this had on the actual study. Respondents could provide multiple answers to these questions. At the end of the survey, respondents had the opportunity to formulate advice to other researchers. Finally, respondents were asked whether they would be willing to provide the relevant documentation about the study they had described, such as the ethics application, the ethics committee’s comments, and their responses to these comments. Respondents who agreed to provide such documents were asked to provide their name and email address. Those who did not do this remained anonymous. The survey was hosted on Strategic Data’s WebSurvey platform (https://strategicdata.com.au/), and all data were saved directly onto a secure server.

### 2.2. Sampling

Eligible respondents had to be actively involved in suicide-related research and able to complete the survey in English. Respondents were recruited through the Australian Suicide Prevention Research Leaders Network. This network comprised 95 researchers working in the suicide prevention field, and was established by our team in March 2018 as part of the NLSPR project. Members of the network received an invitation by email, which included a plain language statement explaining the study and a link to the survey. The landing page of the survey contained consent information, and respondents had to provide online consent before being able to access the survey. Potential respondents received two reminders after the initial invitation. The survey was online for six weeks in the period of September–November 2018, and 33 respondents (35%) completed the survey.

### 2.3. Analyses

All data were uploaded in SPSS version 24 [17]. The quantitative data derived from the forced-choice questions were analysed descriptively and results are presented as frequencies and percentages. The qualitative data from the open-ended questions were analysed using content analysis, which allows for a quantitative and qualitative report of the findings [18]. The content analysis applied a deductive approach (based on the questions in the survey). Two researchers (KA and LR) coded the data through an iterative process, and team discussions with a third researcher (JP) were held to minimize researcher bias.

## 3. Results

### 3.1. Characteristics of the Sample

Table 1 summarizes the characteristics of the sample. There was an almost equal proportion of female and male respondents. On average, respondents had 5–10 years of experience in suicide-related research. They had submitted an average of five applications for suicide-related research studies to ethics committees over the last five years, and on half of these applications they were the principal investigator. Most respondents had received a ‘Minor revision’ request, about half of respondents had received ‘Major revision’ requests, and almost one in ten had ethics applications not approved. Conversely, slightly more than one in ten had ethics applications approved without revisions. A total of 24 respondents named the institution’s ethics committee to which they had submitted most of their ethics applications over the last five years. The institutions responsible for these ethics committees comprised 11 Australian universities (mentioned by 19 respondents), four governmental departments, three hospitals or local health districts and one international health department.

### 3.2. Characteristics of Specific Ethics Applications

**Figure 1:**
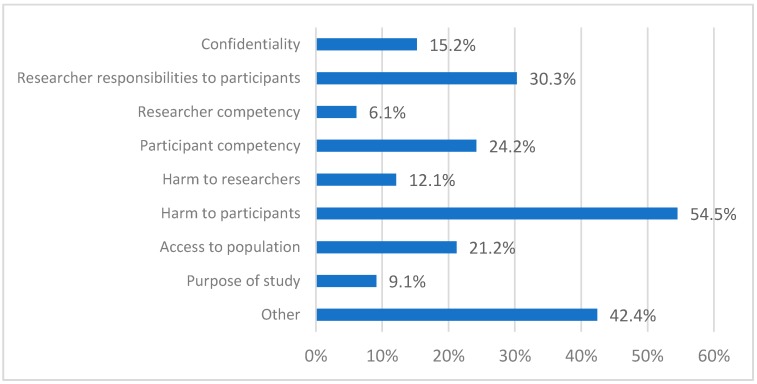
Concerns raised by ethics committees. Total is >100% because multiple answers were allowed.

All respondents provided information regarding one ethics application about which the ethics committee had raised concerns (Table 2). Most respondents categorized their application as an intervention study (for example, a study designed to test the efficacy of a novel approach to improving help-seeking) or evaluation study (for example, a study examining the appropriateness or effectiveness of a particular program or service). The category ‘Other’ included studies on the development of new instruments and qualitative/observational studies. Most applications were focused on young people (45.5%) or adults (30.3%). Regarding the setting, one-third of the applications concerned studies in the community (36.4%) or in a mental health service (36.4%). As multiple answers to these questions were allowed, the totals in Figure 1 are >100%.

Most studies (85%, *n* = 28) involved recruitment of participants; 39% (*n* = 13) of the studies applied quantitative methods, 36% (*n* = 12) mixed-methods and 24% (*n* = 8) qualitative methods.

### 3.3. Concerns Received by Respondents from Their Ethics Committees

Respondents most commonly reported that ethics committees raised concerns in relation to potential harm to participants (Figure 1). According to details provided by respondents, these concerns were related to potential distress or increased risk of suicide due to research participation (33.3%), and/or appropriate management of that distress (18.2%). One-third of the concerns were related to the researchers’ responsibilities to participants. These concerns were primarily focused on the support available to participants (27.3%), and/or resources for participants and non-participants (6.1%). Participant competency was a concern in one in four ethics applications. These concerns were related to participants’ mental capacity due to, for example, mental illness or literacy (12.1%), young age (9.1%) and ability to provide verbal consent (3.0%). The concerns reported in the category ‘Other’ addressed clarifications of definitions and outcome measures (12.1%), project management (12.1%) and data storage (9.1%).

### 3.4. Researchers’ Responses to the Concerns

**Figure 2:**
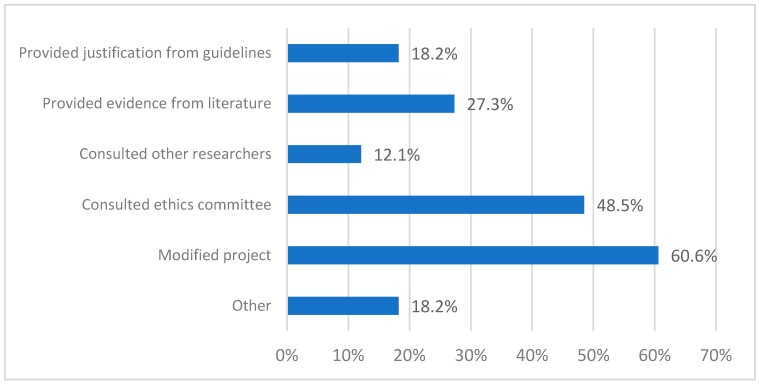
Response to concerns raised. Total is >100% because multiple answers were allowed.

Most researchers modified their ethics application in response to concerns identified by the ethics committee (Figure 2). This involved clarifications and modifications to the study description and participant information (30.3%), recruitment strategy and inclusion and exclusion criteria (15.2%), support available to participants (9.1%) and research method (3.0%). About half of the researchers consulted a representative or the secretariat of their ethics committee to better understand the issues raised (24.2%) and/or to find a consensus (12.1%). The responses listed as ‘Other’ included providing evidence from the literature (12.1%) or from other studies (9.1%).

### 3.5. Impact of the Concerns on the Applications

**Figure 3:**
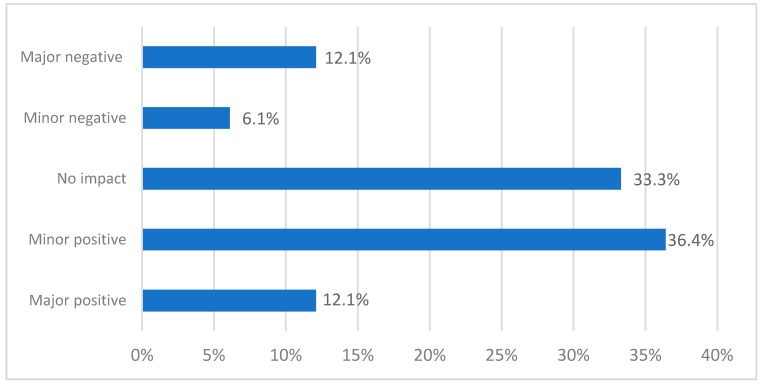
Impact of concerns on the actual study. Total is >100% because multiple answers were allowed.

Most respondents (94%) indicated that they were able to proceed with the study once the concerns had been addressed and their ethics application was approved. About half of the respondents reported that dealing with the concerns had had a positive impact on the actual study, about one in five reported a negative impact and one-third reported no impact (Figure 3). Positive impacts were related to improved clarity in the study design and description (21.2%), a safer study (e.g., better articulated or resourced; 12.1%), valuable experiences for future studies (3.0%) and being able to educate the ethics committee about safe suicide-related research (3.0%). In contrast, negative impacts were related to delays in the start of the study (18.2%) and limitations to recruitment and study sample (6.1%).

The study survey specifically inquired about the duration of the ethics approval process: 67% of respondents indicated that the process took less than 4 months, 9% said it took 5–6 months, and 6% noted that it took 7–9 months. At the other end of the spectrum, 18% of respondents said that the ethics approval process for their selected application took more than 12 months. Thirty-nine per cent of respondents indicated that the duration had caused issues, such as delays in the study (36.4%), additional cost (9.1%) or unavailability of staff (9.1%).

At the end of the survey, all respondents used the opportunity to formulate advice to other researchers. The most common advice was to anticipate possible concerns (36.4%) by providing evidence, such as from the literature, that suicide research can be conducted in a safe way, and by being clear about the study design, possible risks and the procedures to deal with these risks. Or, as one respondent noted: “Put yourself in their shoes. If you were reviewing your own project, what reassurances would you need about consent, safety, confidentiality and consent of what you propose to do?”

In the same vein, respondents recommended that researchers should gain better understanding of how ethics committees work (30.3%), and ‘educate’ their committee, e.g., by providing relevant information and advice (12.1%). Such understanding can be acquired through contacts with members of ethics committees prior or during the ethics approval process, or through experience as a member of such committee. Some respondents (15.2%) highlighted that research experience is helpful, and that senior researchers should not rely on junior researchers to prepare and submit an ethics application or should consider it as a team effort. Finally, respondents recommended that researchers should be patient and include sufficient time in the study design to allow for navigating the ethics process (15.2%). Table A1 (Appendix A) summarizes details of three ethics applications, which were purposefully chosen to illustrate the variety of concerns raised, the researchers’ responses and the impact on the actual study.

## 4. Discussion

This study is the first to investigate concerns that Australian researchers receive from ethics committees in response to suicide-related ethics applications, and how researchers deal with these concerns. Unlike Lakeman and Fitzgerald [13], who reported that researchers had experienced few problems in obtaining ethics approval, possibly due to anticipating potential concerns, respondents to this survey reported substantial concerns. Most of those concerns were related to the well-being of the participants. Potential harm to participants, researchers’ responsibilities to participants and participant competency and consent were the most frequent endorsed categories. This mirrors the concerns mentioned by the members of ethics committees reported by Lakeman and Fitzgerald [14].

In contrast to commonly reported concerns, respondents in our survey reported researcher competency, and potential harm to researchers the least, together with concerns related to the purpose of the study. This finding may indicate that Australian ethics committees are either very sensitive to issues related to safety and well-being of participants, and/or researchers fail to adequately describe these issues in their ethics applications. A substantial number of researchers modified their application, including making changes to the study description, participant information and recruitment strategy. Hence, it is likely that, at least in part, the concerns of the ethics committees were justified. Moreover, the recommendation, formulated by several respondents, that researchers should better anticipate these types of concerns, points in the same direction.

Despite negative impacts experienced by researchers (such as delays, and subsequent costs or problems with staffing and limitations to recruitment), most researchers experienced the impact of the concerns as a minor or major positive impact, or as having no impact. Overall, the reviews of the ethics committees tended to result in improved study designs. Most of the reported ethics committees’ concerns were raised in relation to intervention and evaluation studies. Most of the applications involved recruitment of participants, such as adults or young people, from a community or mental health service setting. Usually, intervention and evaluation studies actively involve participants contrary to epidemiological studies which more commonly rely on routinely collected data [7].

However, while epidemiological studies are the most commonly conducted type of suicide-related study in Australia, intervention studies have been identified as a priority to advance suicide prevention in Australia [16]. Indeed, without intervention and evaluation studies, we will not progress knowledge about what works and what does not work in suicide prevention. Intervention studies are paramount, but our findings indicate that these may present the most important ethical challenges. Hence, researchers and ethics committees need to work together to ensure conduct of safe and high-quality research.

The concerns raised by the ethics committees reported in this study seem to align with the values endorsed by the National Statement on Ethical Conduct in Human Research 2007 (Updated 2018) [15], specifically regarding beneficence and justice. These values are related to potential harm and benefits of the research to participants and the community. And although most researchers modified their applications in response to the concerns, about half of them also contacted their ethics committee. Similarly, many respondents recommended that researchers should better understand the work of ethics committees. This may reflect the National Statement’s notion of a shared responsibility in the ethical design of research studies [15], which endorses our recommendation for further collaboration between researchers and ethics committees.

The literature indicates a lack of standardization in how ethics committees assess ethics applications, suggesting that assessments of similar applications may vary across committees [8]. This observation further underscores the need for a dialogue between researchers and ethics committees. Such a dialogue may concern issues related to the research design, recruitment, consent and so on, but could also address differences in moral views about the extent to which researchers should intervene to prevent a suicide, versus the autonomy of an individual [11]. Mishara and Weisstub [11] distinguished between the moralist view (life must be protected and each suicide stopped), the libertarian view (there is no obligation to intervene to prevent a suicide) and the relativist view (the obligation to intervene depends on the situation, culture and consequences for the person and society). Because such moral stances may influence researchers and members of ethics committees alike, for example, regarding choice of participants, informed consent and participant safety procedures, it is paramount to be aware of one’s own moral attitudes [11]. Clarifying such issues may be necessary to reach a consensus about what constitutes ethically sound research.

### Limitations

Although this study recruited respondents through a national mailing list of suicide-related researchers, it is possible that some relevant researchers were not reached through this list. Thirty-five per cent of those on the list completed the survey. Although it is not possible to determine whether this is a representative sample, it is a typical response rate to this type of study [18]. Still, some caution should be exercised in generalizing study findings to the broader cohort of Australian suicide prevention researchers. A larger number of respondents might have yielded more data, or allowed for more statistical analysis.

The survey also included a few questions regarding the researcher-status of the respondent, and the number of suicide-related ethics applications submitted to ethics committees. Respondents who did not identify as a researcher, or who had not submitted ethics applications, were not able to continue the survey. The survey was conducted online only; some respondents may have preferred other modes of participation. Lastly, the study was conducted in Australia. More international studies are needed to better understand how much of the study findings may apply to other countries.

## 5. Conclusions

Suicide researchers in Australia reported that they have received substantial concerns on ethics applications submitted to their ethics committees. Most concerns were related to potential harm to participants and researchers’ responsibilities to participants. While some researchers experienced a negative impact of dealing with the concerns, most researchers reported a neutral or positive impact. All researchers shared valuable advice to others, such as the importance of anticipating the concerns an ethics committee may raise. Improved understanding of how ethics committees work, and dialogue between researchers and ethics committees should sustain and improve the quality of suicide prevention research, a prerequisite to tackle the increasing suicide mortality in Australia.

## Figures and Tables

**Table 1:** Sample characteristics (*N* = 33).

Variable	Categories or Range	*n* (%) Respondents	Median or *M*, *SD*
Gender	Female	19 (57.6%)	-
	Male	14 (42.4%)	
	Other	0 (0%)	
Years of experience	<5	11 (33.4%)	5–10 years
	5–10	15 (45.5%)	
	>10	7 (21.3%)	
Ethics applications submitted last 5 years (*n* = 180)	Range: 1–25	33	2 5.45 (6.41)
Ethics applications submitted as Principal Investigator last 5 years (*n* = 88)	Range: 1–20	33	1 2.67 (4.02)
Outcomes experienced of all applications submitted last 5 years (*n* = 180) ^1^	Approved	4 (12.1%)	-
	Minor revision	24 (72.7%)	
	Major revision	18 (54.5%)	
	Not approved	3 (9.1%)	

^1^ Total is >100% because respondents could report different outcomes across their ethics applications.

**Table 2:** Characteristics of specific ethics applications.

Variable	Categories	*n* (%) Respondents ^1^
Research type	Social	4 (12.1%)
	Biological	0 (0.0%)
	Evaluation	10 (30.3%)
	Intervention	10 (30.3%)
	Epidemiological	8 (24.2%)
	Assessment	7 (21.2%)
	Other	7 (21.2%)
Focus of the research project	Current or ex-military	1 (3.0%)
	Women	2 (6.1%)
	Men	2 (6.1%)
	Offenders	1 (3.0%)
	People who have attempted suicide	7 (21.2%)
	People with substance use problems	1 (3.0%)
	People with physical health problems	2 (6.1%)
	People with mental health problems	6 (18.2%)
	GLBT+ people	1 (3.0%)
	People bereaved by suicide	6 (18.2%)
	People in rural areas	3 (9.1%)
	Culturally and linguistically diverse people	1 (3.0%)
	Indigenous people	3 (9.1%)
	Older adults	1 (3.0%)
	Adults	10 (30.3%)
	Young people	15 (45.5%)
	Children	0 (0.0%)
	None of these groups	3 (9.1%)
	Other	3 (9.1%)
Setting of the research project	Online	2 (6.1%)
	Mental health service	12 (36.4%)
	Emergency department	1 (3.0%)
	Primary care (e.g., general practice)	2 (6.1%)
	Other health service	3 (9.1%)
	Workplace	0 (0.0%)
	Prison	0 (0.0%)
	Tertiary institution	3 (9.1%)
	School	3 (9.1%)
	Community	12 (36.4%)
	No specific type of setting	5 (15.2%)
	Other	2 (6.1%)

^1^ Totals are >100% because multiple answers were allowed.

## Appendix A

**Table A1:** Summary of sample ethics applications.

	Assessment of Suicide Risk	Group Support after a Suicide Attempt	Feasibility of Online Intervention for Suicidal Ideation
Description	To develop a scale to assess suicide risk, recovery and psychosocial functioning of adults who recently had attempted suicide. Mixed-methods study including focus groups, an online survey providing item set with individuals with lifetime experience of suicide attempt, and testing the scale with individuals with recent and with lifetime history of attempted suicide	Efficacy of a support group for adult persons who had attempted suicide. Mixed-methods study aiming to examine the participants’ suicidality, mood and hopelessness using validated scales, pre and post-test the support group program, and to collect qualitative data from participants and group facilitators.	Single-group pre- and post-test study evaluating an online social-networking-based intervention for young people aged 16–24, who experience suicidal ideation, and who were clients of a tertiary-level mental health service. Mixed-methods study to examine feasibility, safety and acceptability of the intervention, and exploring potential clinical efficacy.
Concerns raised	Potential harm to participants: Asking participants about the relevance of scale items might be distressing or trigger suicidal behaviour. Participant competency: The participant information sheet should draw participants’ attention to their capacity to cope with research participation. Researchers’ responsibilities: Clinicians should actively monitor the focus groups and be available for distressed participants. Confidentiality: Focus group participants might know each other, how to avoid personal disclosures.	Potential harm to participants: Safety of participants exposed to discussions on suicidality and mental health issues. Potential harm to researchers: Impact on researchers conducting detailed interviews with suicidal persons. Participant competency: Whether participants were vulnerable and able to give consent in an objective way. Researchers’ responsibilities: Protocols for responding to participants who seem suicidal or distressed during research participation.	Other concerns: Significance of measuring clinical efficacy in small sample size with no control.
Researchers’ response to concerns	Modified ethics application: Clarified project aims and information about capacity to participate in participant information sheets. Provided additional clinical support for focus groups. Provided evidence from literature: Emphasized extensive literature on low risk of distress from being asked about suicide or mental health in vulnerable populations.	Modified ethics application: Clarified suicide safety and referral protocols. Made consent processes clearer and more explicit. Provided justification: Referred to the body of knowledge on value of lived experience perspectives to research on suicide prevention.	Consulted with ethics committee: Argued that the information would be useful and emphasized that the researchers would not over-interpret the data.
Study proceeded	Yes	Yes	Yes
Impact of concerns	Overall: No impact Negative impacts: greater resourcing requirements, changes in wording and lengthier participant information sheet potentially stigmatizing to participants, and time taken to address comments that were based on misunderstandings. Positive impacts: minor clarifications to participants of nature, risks and study aims.	Minor positive impact. The ethics committee responses focused researchers’ attention on protocols and consent practices, but did not raise issues that they were not already considering. Researchers helped ethics committee to understand that their views of suicidal people were stigmatizing. Duration of ethics process delayed the start of the study.	No impact No changes were needed, only information to justify the design.
Duration of ethics approval process	1–2 months	5–6 months	5–6 months
Advice to other researchers	Cite the literature on distress regarding asking about suicide or mental health. Expect that the ethics committee provide evidence for their assertions. Allow enough time and resources, expect that it will take a while. Push back against stigmatizing responses, educate the committee if possible. Be open to compromise: concessions that do not harm the quality of the research might result in a more favourable outcome.	Provide ethics committees with the rationale and knowledge available on the use of lived experience in research on suicide prevention. Prepare to answer questions around duty of care and consent but reinforce to the ethics committee the importance of not discounting lived experience in research simply because it is somewhat risk laden or difficult to undertake.	Experience, a strong research team and ensuring the study protocol is as thorough as possible. Talk to others who have had success in this area before. Our team has a lot of experience with this kind of projects and was able to pre-empt many of the possible ethical concerns (e.g., around monitoring and managing risk). The team has a good track record of conducting similar research, which definitely helps.
